# Differential Change in Oculomotor Performance among Female Collegiate Soccer Players versus Non-Contact Athletes from Pre- to Post-Season

**DOI:** 10.1089/neur.2020.0051

**Published:** 2020-11-10

**Authors:** Virginia T. Gallagher, Prianka Murthy, Jane Stocks, Brian Vesci, Danielle Colegrove, Jeffrey Mjaanes, Yufen Chen, Hans Breiter, Cynthia LaBella, Amy A. Herrold, James L. Reilly

**Affiliations:** ^1^ Department of Psychiatry and Behavioral Sciences, Northwestern University Feinberg School of Medicine, Chicago, Illinois, USA.; ^2^ Department of Sports Medicine, Northwestern University, Evanston, Illinois, USA.; ^3^ Center for Translational Imaging, Northwestern University, Chicago, Illinois, USA.; ^4^ Division of Orthopedics and Sports Medicine, Ann and Robert H. Lurie Children's Hospital of Chicago, Chicago, Illinois, USA.; ^5^ Edward Hines, Jr. VA Hospital, Hines, Illinois, USA.

**Keywords:** oculomotor, repetitive head impacts, saccade testing, soccer subconcussion

## Abstract

Sensitive and reliable tools are needed to evaluate potential behavioral and cognitive changes following head impact exposure in contact and collision sport participation. We evaluated change in oculomotor testing performance among female, varsity, collegiate athletes following variable exposure to head impacts across a season. Female, collegiate, contact sport (soccer, CONT) and non-contact sport (NON-CONT) athletes were assessed pre-season and post-season. Soccer athletes were grouped according to total season game headers into low dose (≤40 headers; CONT-Low Dose) or high dose (>40 headers; CONT-High Dose) groups. Performance on pro-saccade (reflexive visual response), anti-saccade (executive inhibition), and memory-guided saccade (MGS, spatial working memory) computer-based laboratory tasks were assessed. Primary saccade measures included latency/reaction time, inhibition error rate (anti-saccade only), and spatial accuracy (MGS only). NON-CONT (*n* = 20*)*, CONT-Low Dose (*n* = 17), and CONT-High Dose (*n* = 7) groups significantly differed on pre-season versus post-season latency on tasks with executive functioning demands (anti-saccade and MGS, *p* ≤ 0.001). Specifically, NON-CONT and CONT-Low Dose demonstrated shorter (i.e., faster) anti-saccade (1.84% and 2.68%, respectively) and MGS (5.74% and 2.76%, respectively) latencies from pre-season to post-season, whereas CONT-High Dose showed 1.40% average longer anti-saccade, and 0.74% shorter MGS, latencies. NON-CONT and CONT-Low Dose demonstrated reduced (i.e., improved) inhibition error rate on the anti-saccade task at post-season versus pre-season, whereas CONT-High Dose demonstrated relative stability (*p* = 0.021). The results of this study suggest differential exposure to subconcussive head impacts in collegiate female athletes is associated with differential change in reaction time and inhibitory control performances on executive saccadic oculomotor testing.

## Introduction

Participation in contact and collision sports increases an individual's exposure to head impacts, which may yield concussive or subconcussive injuries. Subconcussive injuries occur in the absence of clinical signs or symptoms, but pathophysiological changes in the brain, including vasculature and white matter alterations, may occur and may have cumulative adverse effects over time.^1–4^ Some studies suggest chronic exposure to repetitive head impacts may be associated with increased risk of long-term neurocognitive deficits and neuropsychiatric disorders.^2,5–7^

Neuroimaging studies evaluating change associated with subconcussive impacts occurring across an athletic season report longitudinal alterations in white matter structure, neurometabolism, and functional activation.^8–15^ Prospective studies examining the relationship between subconcussive impacts and cognitive functioning have yielded mixed findings, with some reporting decline following impact exposure (in domains such as working memory, processing speed, and verbal learning and memory),^15–19^ and others observing no change.^20–26^ Given that cognitive change is seen variably,^7^ it is possible a more sensitive measure is needed to reliably detect subtle changes associated with subconcussive impacts. Saccadic oculomotor testing demonstrates promising sensitivity and specificity for distinguishing individuals with and without exposure to head trauma.^27–30^

Saccades are rapid eye movements from one point to another and can be initiated automatically or voluntarily using top-down executive control.^31^ Laboratory-based saccadic eye movement tasks have been used to probe brain dysfunction as the underlying neural circuitry involved in the execution of saccades is well delineated by human and animal anatomical and physiological research.^31^ Classic saccadic oculomotor tasks include the: 1) pro-saccade task, which measures reflexive shifts of visual attention by prompting participants to look toward a target when it appears; 2) anti-saccade task, which probes inhibition and speed of executive shifts of visual attention by requiring participants to look away from a target when it appears, and 3) memory-guided saccade (MGS) task, which probes working memory ability by prompting participants to look toward a remembered location of a previously presented target after a variable delay period.

A recent meta-analysis revealed that athletes with sports-related concussion perform worse than controls on select aspects of saccade tasks with executive functioning demands, including increased inhibitory errors on anti-saccade and MGS tasks and poorer spatial working memory accuracy on the MGS task.^32^ Further, there is evidence that compared with controls, recently concussed individuals demonstrate longer reaction time on executive saccade tasks, with performance typically normalizing 1 to 2 weeks post-injury.^33–35^ Findings in the literature are inconsistent as to whether oculomotor saccade performance is sensitive to the effects of remote history of concussion.^36–38^ To date, no known studies have investigated the sensitivity of eye movement testing to detect potential effects of recent exposure to subconcussive head impacts sustained across a sports season.

The primary aim of this study was to evaluate whether saccadic oculomotor testing detects differential change in cognitive and sensorimotor processing following variable levels of exposure to head impacts among female contact sport (soccer) and non-contact sport collegiate athletes. Given that executive saccade tasks, such as the anti- and MGS tasks, have been more sensitive to the effects of concussion, relative to the less cognitively demanding pro-saccade task,^32^ we hypothesized that the anti-saccade and MGS tasks would demonstrate sensitivity to the effects of head impact exposure. Specifically, we hypothesized that contact athletes, particularly with higher estimated exposure to head impacts would demonstrate longer anti-saccade and MGS latencies, increased anti-saccade error rate, and reduced MGS accuracy from pre- to post-season (reflecting reduced executive attentional control and working memory ability), compared with non-contact athletes, who would demonstrate relative stability or improvement (reflecting practice effects) over time.

## Methods

### Study design and setting

This prospective, longitudinal study evaluated oculomotor performance among female collegiate athletes assessed pre-season and post-season. Athletes were assigned to the following groups: a) non-contact sport athletes (NON-CONT); b) low-dose contact sport (soccer) athletes who sustained ≤40 headers in games/season (CONT-Low Dose); and c) high-dose contact sport (soccer) athletes who sustained >40 headers in games/season(CONT-High Dose), based on post hoc distribution of header data collected over the season (see Supplementary Fig. S1).

All CONT pre-season assessments were completed prior to the initiation of official pre-season training and at least 4 weeks following the most recent competitive, post-season scrimmage. Post-season procedures occurred within 4–11 days of the end of the competitive season (mean [standard deviation; SD], days = 7.43 [2.50]). NON-CONT athletes were evaluated during similar time frames to match the duration between pre-season and post-season assessments as closely as logistically possible among groups.

### Participants and recruitment

Division 1, female, collegiate varsity athletes (18–25 years) were recruited (*n* = 48) and none met exclusion criteria: history of recent concussion prior to study entry (<30 days),^28–30^ moderate to severe traumatic brain injury,^39^ lifetime diagnosis or family history of a psychotic disorder,^40,41^ seizure disorder,^42^ or vision abnormalities (aside from wearing contacts or glasses for vision correction). The NON-CONT group consisted of golf, swimming, and tennis athletes.

To examine a dose effect of impact exposure among female soccer players, athletes' headers, defined as contact between the player's head and the ball, were recorded live during games by two independent athletic trainers, with any discrepancies resolved by review of game recordings. Headers sustained during practices were not recorded. Based on the distribution of total game headers per season across the CONT group (Supplementary Fig. S1), CONT athletes were divided into CONT-Low Dose (≤40 headers in games/season, *n* = 17) and CONT-High Dose (>40 headers in games/season, *n* = 7) groups.

This study was approved by Northwestern University's Institutional Review Board; all participants completed informed consent procedures prior to participation and were compensated $30 and $45 each for completion of the pre-season study visit and post-season study visit, respectively.

### Oculomotor testing

Eye movement recordings were obtained using the SR Research camera-based eye tracking system, EyeLink 1000 Plus, with a sampling speed of 2000 Hz. Assessments were conducted in a window-less room and a non-restrictive chin and forehead rest stabilized participant head position, maintained a fixed position across subject to visual stimuli, and improved quality of eye trace. Pupil size and corneal reflection thresholds were adjusted for each subject to ensure consistent trace from both eyes. Saccades were automatically detected by the EyeLink system when eye position moved more than 0.15 degrees from fixation and trace exceeded velocity and acceleration thresholds of 30 degrees/sec and 8000 degrees/sec^2^, respectively.

See Table 1 for oculomotor task information. Pro- and anti-saccade tasks consisted of gap, no gap, and overlap trials. On no gap trials, the central target extinguished simultaneously with the peripheral target appearance, whereas the central target was extinguished 200 msec prior to, or following, the peripheral target appearance on gap and overlap trials, respectively. The order of condition administration within the pro- and anti-saccade tasks was randomized by participant but was consistent within participant at both visits.

**Table 1.** Oculomotor Task Information

Task (duration)	Overview	Primary measure(s)	Directions to participant	Design
Pro-saccade (10 min)	An automatic or reflexive attention task.	Primary saccade latency, gain, accuracy, duration, and peak velocity.	Participants are instructed to look at visual targets when they appear.	96 trials that begin with a center fixation target that remains illuminated for 1.5–2.5 sec before peripheral targets appear at 10 degrees or 15 degrees from center in the left or right visual field; three conditions (32 trials each) are conducted to manipulate the center fixation offset and peripheral target onset: gap, no gap, and overlap.
Anti-saccade (10 min)	An executive or voluntary attention task.	Antisaccade error rate (error trials/total trials) and primary saccade latency. Errors occur when participants incorrectly look toward the peripheral target.	Participants are instructed to inhibit the automatic response to look toward the peripheral target and instead shift their gaze to the mirrored location (e.g., if target appears 3 in to the left of center, participants look immediately 3 in to the right of center).	96 trials that begin with a center fixation target that remains illuminated for 1.5–2.5 sec before peripheral targets appear at 10 degrees or 15 degrees from center in the left or right visual field; three conditions (32 trials each) are conducted to manipulate the center fixation offset and peripheral target onset: gap, no gap, and overlap.
Memory-guided saccade (20 min)	An executive, working memory task.	Primary saccade latency, spatial accuracy, and delay duration error rate (errors occur when the participant incorrectly looks toward the target during the delay period).	Participants are instructed to maintain gaze on the central fixation stimulus; after a short delay, a peripheral target appears very briefly as participants continue to fixate on the central stimulus. When the central fixation stimulus disappears after a brief delay, participants shift their gaze to the remembered location of the peripheral target.	80 trials that begin with a center fixation target that remains while the peripheral target (at 6 degrees, 8 degrees, 10 degrees, 12 degrees, and 14 degrees from center in the left or right visual field) appears and extinguishes with variable delay duration period (1, 2, 4, 8 sec).

Primary variables of interest included saccade latency, defined as the time (in milliseconds) between the cue to respond and the initiation of a saccade, which reflects the speed of reflexive shifts of visual attention on the pro-saccade task and the speed of executive or voluntary shifts of attention on the anti-saccade and MGS tasks. On the pro-saccade task, in addition to latency, other measurements of interest included: a) gain: the spatial accuracy of the saccade to the target location, which is derived from the ratio of the saccade amplitude to the target amplitude; b) accuracy: the spatial accuracy of the primary saccade, derived from the difference between the location of the target position and the location of the end point of the primary saccade (in x-coordinate pixels); c) duration: the time (in milliseconds) taken to complete a saccade; and d) peak velocity (degree/sec): the highest velocity reached during the saccade, which is linearly related to the saccade duration. Anti-saccade error rate, the ratio of error trials (in which a participant incorrectly looks to a target when it appears) to total trials, is a measure of executive response inhibition and cognitive control. MGS accuracy, the percentage of distance (overshoot or undershoot) of the saccade to the remembered location, is a measure of spatial working memory precision.

### Questionnaires and self-reported symptom data

Prior to in-person study assessments, all participants completed a comprehensive questionnaire regarding demographic, health history (including concussion history), recent athletic participation information, educational background, and parental education and occupational history (to yield a total socioeconomic status score).

All athletes completed several online self-report questionnaires within 24 h of eye movement testing at both visits to assess the influence of physical, cognitive, and emotional symptoms on oculomotor performance. Questionnaires included the Post-Concussion Symptom Scale (PCSS) to assess the severity of physical, cognitive, and emotional symptoms that occur in both concussed and healthy populations;^43^ the Beck Depression Inventory-II (BDI-II) to assess symptoms of depression;^44,45^ the State-Trait Anxiety Inventory (STAI) to assess anxiety,^46^ and the Perceived Stress Scale (PSS) to assess the ability to handle stress.^47,48^

### Participation data

Senior athletic trainers recorded participant attendance and participation in practices and games to ensure adequate engagement in athletics and no prolonged (>14 day) absences due to injury. To ensure comparable exposure to recent exercise and competition at the post-season visit, the duration between the dates of most recent sports participation and post-season assessments were calculated for each player and compared among groups.

### Statistical analysis

See eMethods in Supplementary Appendix S1 for oculomotor data scoring and cleaning procedures established in our laboratory.^49^ Statistical analyses were performed using IBM SPSS Statistics for Windows, version 25.0.^50^ Descriptive analyses were conducted to evaluate group differences in demographic, clinical history information, and mood/behavioral symptom data; specific tests included one-way analyses of variance and chi-square tests for parametric data and Kruskal-Wallis analyses for non-parametric data. General linear mixed modeling was used to evaluate group differences on oculomotor measures of interest within task. For pro-saccade latency, anti-saccade latency (correct trials only), and anti-saccade error rate, effects analyzed included visit (pre-season versus post-season), group (NON-CONT vs. CONT-Low Dose vs. CONT-High Dose), condition (gap, no gap, overlap), the two-way interaction of visit-by-group, and the three-way interaction of visit-by-group-by-condition. Given that the experimental manipulation of the offset of the central fixation target primarily affects latency and anti-saccade error rate, all other pro-saccade analyses (gain, accuracy, duration, and peak velocity) were conducted on no gap condition trials only. For MGS latency and accuracy of the primary saccade and resting position, effects analyzed included visit, group, delay period duration (1-, 2-, 4-, or 8-sec delay period duration), the two-way interaction of visit-by-group, and the three-way interaction of visit-by-group-by-delay period duration.

If an effect was not significant (*p* > 0.05) in the model, it was removed from the model, and main effects of interest were retained (i.e., visit-by-group or visit-by-group-by-condition) for a repeat analysis. Multiple repeated covariance structure types were tested for each model, and the repeated covariance structure with the lowest Akaike Information Criteria (AIC), indicating the best model fit, was selected for final analysis.^51^ The original alpha level (*p* < 0.05) was adjusted for multiple comparisons using the Bonferroni correction across the four main hypotheses to yield a new alpha level of *p* < 0.0125.

## Results

See Table 2 for sample characteristics among NON-CONT (*n* = 20), CONT-Low Dose (*n* = 17), and CONT-High Dose (*n* = 7). Two participants ([*n* = 1, CONT-Low Dose] and [*n* = 1, NON-CONT]) from the original sample were lost to follow-up. One NON-CONT athlete was excluded post hoc due to in-season orthopedic injury, and one NON-CONT was excluded due to invalid performance. Participant dropout and exclusion rates did not differ among groups. One CONT-High Dose athlete sustained a concussion 18 days prior (with medical clearance 9 days prior) to the post-season visit. This participant's oculomotor measures were within ±1 SD of the CONT-High Dose group means. Results of analyses remained consistent with and without inclusion of this participant; as such, this participant was retained. Groups did not differ on the average number of days between most recent game or practice and the post-season visit. Groups significantly differed by history of previous concussion and history of learning disability and/or attention-deficit/hyperactivity disorder (ADHD); none of these variables were associated with oculomotor performance at baseline or change over time. There were no significant differences in self-reported symptoms of depression, anxiety, stress, or concussion-related symptoms at pre-season or post-season, nor were there were significant group-by-time interaction effects (see Table 3).

**Table 2.** Sample Demographics and Characteristics

	NON-CONT (*n* = 20)	CONT-low dose (*n* = 17)	CONT-high dose (*n* = 7)	P-value
Age, mean (SD), years	19.50 (1.40)	19.24 (1.44)	20.14 (1.21)	ns
Caucasian (%)	13 (65%)	14 (82%)	7 (100%)	ns
Years of education, mean (SD)	13.35 (1.18)	12.71 (1.05)	13.43 (0.98)	ns
Socioeconomic status, mean (SD)^a^	150.00 (24.58)	141.00 (23.82)	149.33 (10.01)	ns
Hx of anxiety or depression (%)	2 (10%)	2 (12%)	2 (29%)	ns
Hx of LD or ADHD (%)^b^	1 (5%)	0 (0%)	2 (29%)	0.038
Hx of 1+ previous concussion^b^	0 (0%)	7 (41%)	4 (57%)	0.002
Weeks between pre- and post-season visits, mean (SD)	18.00 (3.67)	20.59 (14.11)	19.71 (2.98)	ns

^a^ Represents Hollingshead 4-factor score; ^b^despite significant group differences, variable was not associated with oculomotor performance at baseline or change over time.

ADHD, attention-deficit/hyperactivity disorder; Hx, History; LD, learning disability; ns, not significant *(p* > 0.05); SD, standard deviation.

**Table 3.** Group Mood and Symptom Data

		NON-CONT	CONT-low dose	CONT-high dose
		(*n* = 20)	(*n* = 17)	(*n* = 7)
Pre-season mean (SD)			
	BDI-II	3.65 (4.86)	2.82 (3.34)	3.57 (3.64)
	PCSS	12.05 (10.79)	10.00 (11.03)	13.43 (11.27)
	STAI-State	32.35 (8.25)	33.88 (8.00)	37.14 (6.04)
	STAI-Trait	37.20 (10.14)	35.41 (7.68)	37.29 (10.39)
	PSS	14.25 (5.37)	13.94 (4.85)	12.57 (4.08)
Post-season mean (SD)				
	BDI-II	4.50 (6.72)	3.35 (3.89)	5.00 (3.83)
	PCSS	8.85 (10.85)	7.41 (6.56)	9.57 (13.84)
	STAI-State	35.35 (11.74)	35.00 (9.16)	35.71 (10.45)
	STAI-Trait	34.10 (10.47)	36.71 (9.57)	35.43 (11.03)
	PSS	15.10 (7.24)	15.94 (5.80)	14.71 (5.82)

There were no significant differences among groups in self-reported symptoms of depression, anxiety, stress, or concussion-related symptoms at pre-season or post-season, nor were there significant group-by-time interaction effects.

BDI-II, Beck Depression Inventory-II; PCSS, Post-Concussion Symptom Scale; PSS, Perceived Stress Scale; SD, standard deviation; STAI, State-Trait Anxiety Inventory.

Regarding position, the CONT-Low Dose group was comprised of approximately 47% midfielders, 24% forwards, 18% goalkeepers, and 12% defenders and the group average of game playing time was 495 min (SD = 470). The CONT-High Dose group was comprised of approximately 57% midfielders and 43% defenders with a group average of 1594 min (SD = 156) of game playing time. Although we did not have header data for practices, it is reasonable to assume players in the CONT-High Dose group sustained an overall greater frequency of head impacts throughout the season given data in the literature suggesting female college soccer players sustain two times the frequency of head impacts during games versus practices.^52^ Further, the distribution of positions across the groups is not surprising given data demonstrating higher mean header exposures among midfielders and defenders relative to forwards and goal-keepers.^53^ Across the season, the CONT-Low Dose group sustained an average of 8.94 headers in games (SD = 10.04; range 0–34) whereas the CONT-High Dose group sustained an average of 70.71 headers in games (SD = 24.5, range = 48–115).

### Pro-saccade task

**FIG. 1.**
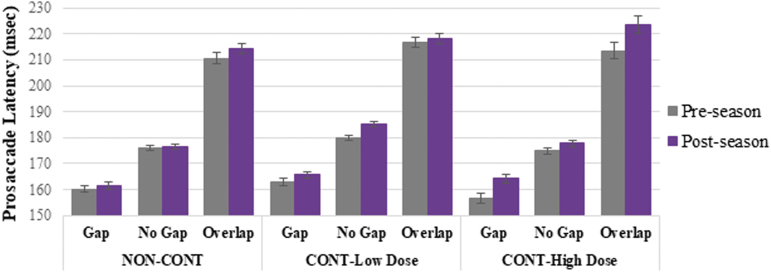
Pro-saccade latency by visit, group, and condition. Error bars represent standard error of the mean. Compared to pre-season performance, CONT-High Dose trended toward longer latency at post-season particularly on gap and overlap conditions, to a greater degree than CONT-Low Dose and NON-CONT (visit-by-group-by-condition interaction effect, *p* = 0.082).

When interpreting change over time on oculomotor measures, shorter latency at post- versus pre-season is reflective of faster reaction time, or improved performance. Decreased anti-saccade error rate at post- versus pre-season reflects improved performance. Analysis of reflexive, pro-saccade task performance from pre- to post-season revealed a trending visit-by-group-by-condition interaction effect on latency (*p* = 0.082), such that CONT-High Dose demonstrated longer average latency at post-season, relative to pre-season, particularly on gap (4.90% increase) and overlap (3.77% increase) conditions, whereas CONT-Low Dose demonstrated more modest increases in latency (2.95% on no gap, 1.77% on gap), and NON-CONT demonstrated a modest increase in latency on overlap trials only (1.81% increase, <1% change on other trials); see Figure 1. There were no significant visit-by-group effects on pro-saccade gain, accuracy, or duration. There was a significant (*p* = 0.018) visit-by-group interaction on peak velocity (no gap condition only) in which NON-CONT demonstrated a 2.94% decrease, CONT-Low Dose demonstrated a 0.64% decrease, and CONT-High Dose demonstrated a 4.42% decrease from pre- to post-season. Notably, CONT-High Dose demonstrated faster peak velocity at pre-season assessment (mean [standard error of the mean; SEM], degrees/sec, 407.05 [3.87]) relative to pre-season peak velocity of CONT-Low Dose (384.81 [2.72]) and NON-CONT (391.43 [2.63]) groups.

### Anti-saccade task

**FIG. 2.**
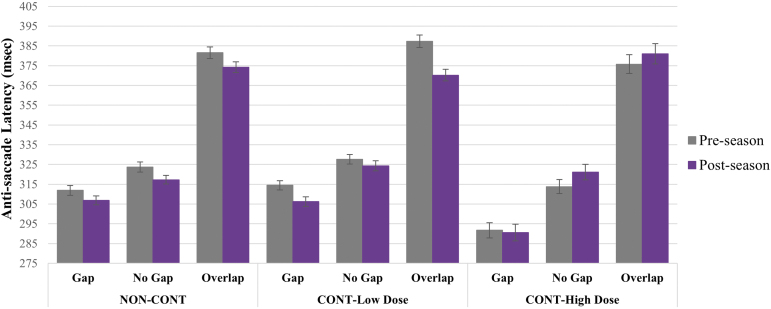
Anti-saccade latency by visit, group, and condition. Error bars represent standard error of the mean. Compared with pre-season performance, NON-CONT demonstrated shorter post-season latency to a greater degree than CONT-Low Dose, whereas CONT-High Dose demonstrated longer post-season versus pre-season latency on no gap and overlap conditions (visit-by-group-by-condition effect, *p* = 0.001).

**FIG. 3.**
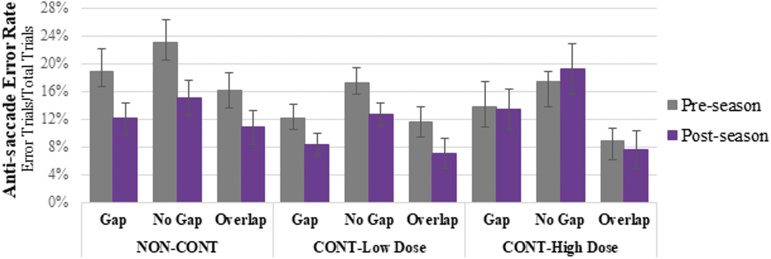
Anti-saccade error rate by visit, group, and condition. Error bars represent standard deviation. Compared with pre-season performance, NON-CONT and CONT-Low Dose demonstrated greater reduction in anti-saccade error rate at post-season compared with CONT-High Dose (*p* = 0.021).

There were significant visit-by-group-by-condition *(p* = 0.001) and visit-by group *(p* < 0.001) effects on anti-saccade latency in which NON-CONT and CONT-Low Dose demonstrated shorter anti-saccade latencies from pre- to post-season, whereas CONT-High Dose demonstrated longer latencies, with differential effects observed across conditions (Fig. 2). There was also a significant visit-by-group, although no visit-by-group-by-condition, effect on anti-saccade error rate (*p* = 0.021); however, the effect does not survive the Bonferroni correction *(p* > 0.0125). From pre- to post-season averaged across conditions, NON-CONT demonstrated an average of 34.77% reduction in error rate (mean [SD] pre-season: 19.32% [13.81%], post-season: 12.60% [10.89%]) and CONT-Low Dose a 31.83% reduction (pre-season: 13.66% [8.86%], post-season: 9.31% [7.89%]), but CONT-High Dose remained stable (<1.00% change); pre-season: 13.39% [7.07%], post-season: 13.39% [9.28%]); see Figure 3.

### Memory-guided saccade task

**FIG. 4.**
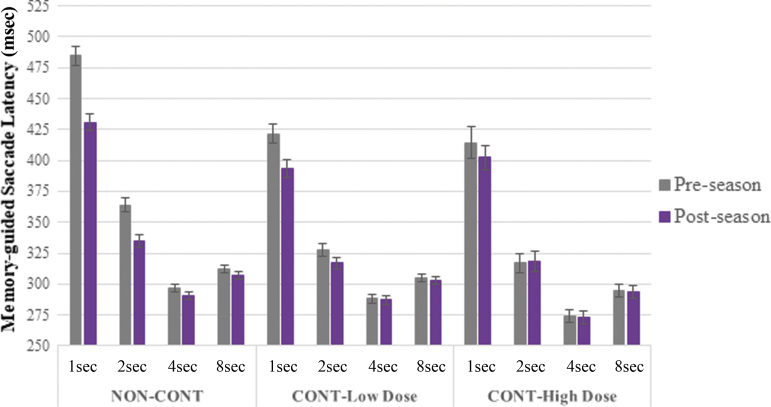
Memory-guided saccade latency by visit, group, and condition (delay period interval). Error bars represent standard error of the mean. NON-CONT demonstrated 5.74% shorter, CONT-Low Dose 2.76% shorter, and CONT-High Dose 0.79% shorter average post-season versus pre-season latency, with greater effects observed on the 1-sec and 2-sec delay conditions (visit-by-group-by-condition and visit-by group effects, *p* < 0.001).

There were significant visit-by-group-by-delay interval *(p* < 0.001) and visit-by group *(p* < 0.001) effects on MGS latency with groups showing pre-season to post-season improvement in a stepwise fashion such that NON-CONT demonstrated shorter pre- to post-season latencies followed by CONT-Low Dose and CONT-High Dose, which showed minimal change (5.74%, 2.76%, and 0.79%, respectively, averaged across delays). This pattern is particularly evident on shorter delay period durations (i.e., 1- and 2-sec delay durations); see Figure 4. There were no significant visit-by-group-by-condition or visit-by group effects on MGS spatial accuracy of the primary saccade or the final resting eye position.

## Discussion

In this study evaluating the use of oculomotor testing to detect differential change in cognitive and sensorimotor processing following variable exposure to head impacts over the course of a competitive season, we found that non-contact sport female athletes and female soccer players with relatively lower header exposure demonstrated faster oculomotor reaction time from pre- to post-season, likely attributable to practice effects, on tasks with executive functioning demands (namely, attentional control/inhibition and spatial working memory), whereas female soccer players with relatively higher header exposure remained stable or subtly declined from pre- to post-season. Similarly, compared with pre-season performance, non-contact sport athletes and soccer players with relatively lower header exposure demonstrated improvement in response inhibition, reflected by reduced anti-saccade error rate, at post-season, whereas female soccer players with relatively higher header exposure remained stable. There were no significant differences among groups in change over time on reflexive, pro-saccade reaction time, suggesting increased sensitivity of tasks with executive functioning demands (anti-saccade and MGS) to the effects of head impact exposure. This is not surprising given that athletes with recent concussion do not reliably demonstrate altered pro-saccade reaction time.^32^

Multiple neuroimaging methodologies have detected adverse change from pre- to post-season among collision sport athletes, with change attributable to exposure to subconcussive head impacts that yield no clinical symptoms.^9,11–13,15,54–56^ However, prospective investigations of behavioral and/or cognitive functioning from pre- to post-season have produced mixed results.^16,18–20,22–26^ The results of this study suggest that oculomotor testing may possess adequate sensitivity to distinguish differential patterns of change in sensorimotor and cognitive functioning following variable exposure to impacts over the course of a female collegiate soccer season. Further, our findings were on tasks of executive functioning, which is consistent with neuroimaging literature citing alterations in neurometabolism, functional activation, and connectivity in the dorsolateral prefrontal cortex (DLPFC) as a result of subconcussive impacts.^8,15,57^

In our primary hypotheses, we predicted that CONT-High Dose athletes would demonstrate longer anti-saccade and MGS latencies, whereas CONT-Low Dose and NON-CONT would demonstrate stability or improvement over time. The direction of our hypotheses was supported in that the NON-CONT and CONT-Low Dose groups demonstrated improved performance and CONT-High Dose demonstrated a subtle decline (anti-saccade latency) or remained stable (MGS latency). Greater improvement in anti-saccade latency over time among CONT-Low Dose (2.68% shorter) relative to NON-CONT (1.84% shorter) was surprising given that we expected athletes with any exposure to head impacts to demonstrate more adverse change relative to athletes with no exposure. This finding may be explained by marginally faster average pre-season anti-saccade latency among NON-CONT (mean [SEM], msec = 339.06 [2.62]) versus CONT-Low Dose (343.12 [2.64]), thus creating greater opportunity for improvement in the CONT-Low Dose group at post-season. The CONT-High Dose group had the fastest pre-season average anti-saccade latency (327.08 [4.07]) among the three groups, which may suggest the subtle decline in performance observed from pre-season to post-season is partially due to a ceiling effect in which the opportunity for significant improvement from baseline is limited. Nonetheless, a lack of improvement from pre- to post-season among CONT-High Dose may reflect subtle adverse effects of exposure to increased headers on speed of voluntary responding, which is consistent with findings of prolonged anti-saccade latencies among participants following concussion.^28,34,58,59^

With regard to MGS latency, NON-CONT demonstrated 5.74% shorter, CONT-Low Dose 2.76% shorter, and CONT-High Dose 0.79% shorter post-season latency (averaged across conditions) compared with pre-season latency, with effects particularly apparent on trials with shorter delay durations. Similar to those on anti-saccade latency, the effects observed on MGS latency may be partially due to better performance at baseline among CONT groups (MGS latency mean [SEM], msec: CONT High Dose = 325.14 [7.72], CONT Low Dose = 335.71 [4.91]) relative to NON-CONT (364.48 [4.98]), leaving more opportunity for improvement among NON-CONT. However, the greater improvement among CONT-Low Dose versus High Dose suggests differential change may be partially attributable to greater relative estimated exposure to head impacts among CONT-High Dose. If differential change among groups on anti- and MGS latencies from pre- to post-season can be at least partially ascribed to varied exposure to head impacts, it suggests the cortical regions, or connections among them and their projections to subcortical areas involved in response latency of voluntary saccadic responses, including the frontal eye fields (FEF),^60–62^ DLPFC,^63^ supplementary eye fields (SEF), anterior cingulate, and intraparietal sulcus,^60^ may be vulnerable to the effects of subconcussive impacts.

In our primary hypotheses, we also predicted that CONT-High Dose would demonstrate increased anti-saccade error rate, compared with CONT-Low Dose and NON-CONT, who would show relative stability or improvement over time. Indeed, NON-CONT and CONT-Low Dose groups demonstrated similar improvement from pre- to post-season (>30% reduction in error rate). However, CONT-High Dose remained stable over time, although this analysis did not survive the Bonferroni correction for multiple comparisons. Nonetheless, the lack of improvement from pre- to post-season among the CONT-High Dose group could be ascribed to greater estimated exposure to head impacts because contact groups had similar pre-season performances (anti-saccade error rate, mean [SD]: CONT High Dose = 13.39% [7.07%], CONT-Low Dose = 13.66% [8.86%]) and therefore, results cannot be attributed to a ceiling effect.

The discrepant change in error rate from pre- to post-season among CONT-High Dose relative to NON-CONT and CONT-Low Dose groups is also consistent with the post-concussion literature that demonstrates higher anti-saccade error rates among participants with recent concussion versus controls.^28,34,59,64^ Therefore, the regions and their connections involved in the execution of anti-saccades—including frontal areas (particularly DLPFC, medial FEF, SEF, and middle frontal gyrus [MFG]), parietal sensorimotor areas (inferior parietal lobule), to a lesser extent subcortical areas (anterior cingulate), and the cortico-striatal pathway from the FEF to the superior colliculus (SC) involving the caudate nucleus—may be affected by exposure to subconcussive and concussive hits.^65–72^ Recent evidence suggests head trauma may disrupt proper functioning of cortico-striatal projections, which supports the suggestion that this mechanism may underlie the group effects observed on anti-saccade error rate.^73,74^

Our hypothesis that CONT groups would demonstrate differential change on MGS spatial accuracy, a measure of spatial working memory ability, relative to NON-CONT from pre- to post-season, was not supported. Given that differential change was observed among groups with varying levels of estimated exposure to head impacts on anti-saccade error rate but not on MGS spatial accuracy, it is possible that inhibitory control abilities may be more susceptible to the effects of head impact exposure relative to spatial working memory.

Overall, we detected differences over time in performance on tasks with executive functioning demands, and not on a reflexive sensory task, among athletes with higher levels of estimated exposure to head impacts over the course of a season. Specific executive functioning demands included executive control and reaction time on tasks of inhibition and working memory. Investigations of the effects of subconcussive head impacts on executive functioning and processing speed using traditional or computerized neuropsychological measures have generally not revealed significant findings.^75^ This suggests that eye movement testing may have greater sensitivity to subtle differences in executive functioning changes. Further, taking together neuroimaging evidence revealing altered structure and function in DLPFC following subconcussive impact exposure and the known role of DLPFC in the execution, and to some extent, speed of anti-saccades, the results of our study suggest subconcussive impacts may affect functioning associated with DLPFC.^8,57,61,71,76^

## Conclusion

In summary, NON-CONT and CONT-Low Dose groups both demonstrated improved performance, likely attributable to practice effects, from pre-season to post-season on oculomotor measures of reaction time and attentional control, whereas CONT-High Dose did not show improvement on these measures. Differential changes were not attributable to variations in mood, somatic, or physical symptoms at times of assessment or pre-existing history of concussion, ADHD, or learning disability (see Tables 2 and 3). Therefore, the lack of improvement among the CONT-High Dose athletes, with the greatest estimated exposure to head impacts over the course of the season, may be interpreted as a negative consequence of elevated exposure to head trauma. Although the aforementioned effects were statistically significant, the magnitude of effects was quite small, so likely not clinically significant. This is not surprising given that the CONT-High Dose group did not report increased concussion-related physical, somatic, mood, or sleep symptoms from pre- to post-season (Table 3). The similar change in performance over time between non-contact sport athletes and female soccer players with relative lower exposure to headers suggests a dose-dependent effect, where lower levels of exposure to head impacts may not yield adverse change over time in terms of oculomotor reaction time and attentional control.

### Limitations

Certain study limitations warrant mention. First, although we were able to quantify headers incurred during games, we did not quantify soccer headers sustained during practice sessions nor were we able to collect information regarding the magnitude and location of head impacts. It is likely, and anecdotally reported by the senior athletic trainers, that players who sustain more headers in games also sustain more headers in practices, but we lack quantitative data to support that claim. Further, players may have sustained non-header head impacts (e.g., collision with other players) that are not accounted for. Second, it is possible that female athletes who engage in “ball” sports may have faster reaction times in response to external stimuli; our control, non-contact athlete group consisted of tennis, golf, and swimming athletes, whose performance on our tasks may be influenced by the unique oculomotor and reaction time demands of their respective sport. This could be addressed in future studies that restrict the control group to only ball or non-ball sports. Third, pre-existing visual disturbances were not evaluated outside of participant self-reported problems with vision. Future directions of this research include expanding the protocol to a larger sample size, adding follow-up oculomotor assessments to evaluate the extent to which disparate performances from pre- to post-season may normalize after a period of rest from exposure to subconcussive hits, and expansion to youth, professional, and male athletes.

## Supplementary Material

Supplemental data

## Abbreviations Used

ADHD: attention-deficit/hyperactivity disorder
AIC: Akaike Information Criteria
BDI-II: Beck Depression Inventory-II
CONT-High Dose: athletes who sustained >40 headers in games/season
CONT-Low Dose: athletes who sustained ≤40 headers in games/season
DLPFC: dorsolateral prefrontal cortex
FEF: frontal eye fields
Hx: History
MFG: middle frontal gyrus
MGS: memory-guided saccade
NON-CONT: non-contact sport athletes
PCSS: Post-Concussion Symptom Scale
PSS: Perceived Stress Scale
SD: standard deviation
SEF: supplementary eye fields
SEM: standard error of the mean
STAI: State-Trait Anxiety Inventory
