# Supplementary material for: Differential Change in Oculomotor Performance among Female Collegiate Soccer Players versus Non-Contact Athletes from Pre- to Post-Season
Source: Neurotrauma Rep. 2020 Nov 10;1(1):169–80. doi: 10.1089/neur.2020.0051 (PMC7703496; doi:10.1089/neur.2020.0051)

**Online Supplementary Material**

**Supplementary Appendix S1**

**eMethods**

**Oculomotor data scoring and cleaning procedures.** Oculomotor data from the right eye only were exported using the EyeLink Data Viewer software package (SR Research Ltd., version 3.1). For all tasks, primary saccades were defined as the first saccade after the target appearance that originated within ± two visual degrees from center and amplitude ≥ 20% of the absolute distance toward the target measured in degrees of visual angle. Primary saccades were excluded from analyses if a blink occurred within 100 ms prior to the appearance of the target. If a primary saccade contained a blink, the latency information was retained for analyses, but all other saccade measurements of interest were excluded from analyses. Unless otherwise stated, all analyses of oculomotor measurements of interest were conducted on the primary saccade of the trial. Resulting saccade measurements were trimmed to exclude values below approximately the 2.5 percentile and above approximately the 97.5 percentile values across the sample to normalize the distribution of data; in addition, cut-off values were set after incorporating qualitative review of the frequency histograms with consensus among at least two research team members.

On the antisaccade task, an error trial occurs when the participant incorrectly looks ≥ 20% of the distance toward the peripheral target. On the memory-guided saccade task, primary saccades that were preceded by a stimulus-driven error during the delay period (i.e., a look to the target within 120–300 ms after target appearance) were excluded from latency and accuracy analyses.

**Supplementary Fig. S1**. Distribution of Total Headers in Games Per Season Across Contact Athletes


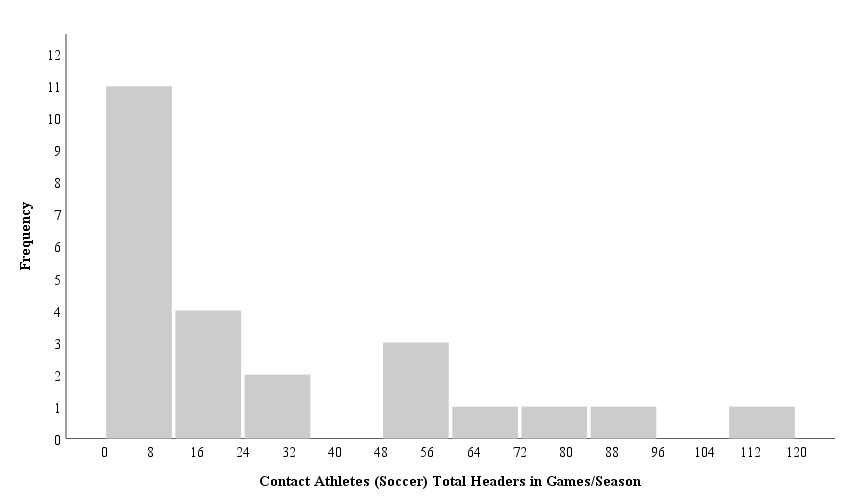

Supplement: Supplemental data [file Supp_Data.docx]
